# Fabrication and thermoelectric conversion of thermoelectric concrete brick with buried unileg N-type CaMnO_3_ thermoelectric module inside

**DOI:** 10.1038/s41598-023-28080-7

**Published:** 2023-01-17

**Authors:** Keerati Maneesai, Sunisar Khammahong, Pongsakorn Siripoom, Chaiwat Phrompet, Chaval Sriwong, Santi Maensiri, Chesta Ruttanapun

**Affiliations:** 1 Department of Physics, School of Science, King Mongkut’s Institute of Technology Ladkrabang, Chalongkrung Road, Ladkrabang, Bangkok, 10520 Thailand; 2 Smart Materials Research and Innovation Unit, School of Science, King Mongkut’s Institute of Technology Ladkrabang, Chalongkrung Road, Ladkrabang, Bangkok, 10520 Thailand; 3 Center of Excellence in Smart Materials Research and Innovation, King Mongkut’s Institute of Technology Ladkrabang, Chalongkrung Road, Ladkrabang, Bangkok, 10520 Thailand; 4 Thailand Center of Excellence in Physics, Ministry of Higher Education, Science, Research and Innovation, 328 Si Ayutthaya Road, Bangkok, 10400 Thailand; 5 School of Physics, Institute of Science, Suranaree University of Technology, Nakhon Ratchasima, 30000 Thailand; 6 Department of Chemistry, School of Science, King Mongkut’s Institute of Technology Ladkrabang, Chalongkrung Road, Ladkrabang, Bangkok, 10520 Thailand; 7 College of Innovation and Industrial Management, King Mongkut’s Institute of Technology Ladkrabang, Chalongkrung Road, Ladkrabang, Bangkok, 10520 Thailand

**Keywords:** Energy science and technology, Materials science

## Abstract

To investigate the effect of heat loss reduction due to thermal insulator and thermal interface resistance due to multi-layer structure in order to improve the efficiency of a thermoelectric device, a thermoelectric concrete brick was fabricated using a unileg n-type CaMnO_3_ thermoelectric module inside. CaMnO_3_ thermoelectric materials were synthesized by starting materials CaCO_3_ and MnO_2_ to produce a unileg n-type CaMnO_3_ module. Thermoelectric concrete brick consisted of two types: I-layer brick (one layer of concrete thermal insulator) and III-layer brick (three layers of different concrete insulators). The occurring temperature difference, electric current and voltage on the CaMnO_3_ module and thermoelectric concrete brick were measured in closed and open circuits. The temperature difference, thermal distribution, and output voltage when applying constant temperatures of 100, 200 and 400 °C were measured. Computer simulations of the Finite Element Method (FEM) were performed to compare with the experimental results. The trends of the temperature difference and the output voltage from the experimental and computer simulations were in good agreement. The results of the temperature difference during the hotter side temperature of 200 °C exhibited the temperature difference along the vertical direction of the thermoelectric concrete bricks for both types of the III-layer brick of 172 °C and the I-layer brick of 132 °C are larger than that of the CaMnO_3_ TEG module without using a thermal concrete insulator of 108 °C. The thermoelectric concrete bricks of the III-layer brick type of 27.70 mV displayed output voltage results being higher than those of the I-layer brick of 26.57 mV and the CaMnO_3_ TEG module without using a thermal concrete insulator of 24.35 mV. Thermoelectric concrete brick of the III-layer brick type displayed higher electric generation power than the I-layer brick and the CaMnO_3_ TEG module. Additionally, the results exhibited the capability of thermoelectric concrete brick in the III-layer brick model for electric generation power based on the temperature difference. The TEG concrete brick of I-layer concrete covering the series–parallel combination circuit of 120 modules of the unileg n-type CaMnO_3_ was constructed and then embedded on the outer surface of the furnace. During the maximum hotter side temperature of 580 °C of the concrete brick, the temperature difference between the hotter side and the cooler side of the brick occurred at 365 °C and the maximum output voltage was obtained at 581.7 mV.

## Introduction

Thermoelectric Generators (TEGs) are devices generating electrical power directly from thermal energy. TEGs can work without mechanically moving parts and nonchemical reactions as the advantage of TEGs is no pollution and silencing^1^. Applications of TEG include electricity generation in space and remote areas, waste heat recovery in automobiles and industries, microelectronics and sensors^2^, biomass stoves^3,4^, solar thermoelectric generator (STEG)^5^, textile^6^, paint^7^ and wearable thermoelectric devices^8–11^.

The thermoelectric conversion efficiency of the TEG (η) is defined as the ratio between the electric output power (P) delivered to the load and the rate of heat input (Q̇_h_) absorbed at the hot junction of the TEG using the following equation $\eta = \frac{P}{{\dot{Q}_{h} }}$^12^: The TEG efficiency is also calculated in form of materials figure of merit by^13^:

1 $$\eta = \frac{{T_{H} - T_{C} }}{{T_{H} }}\left[ {\frac{{\sqrt {1 + ZT} - 1}}{{\sqrt {1 + ZT} + \frac{{T_{C} }}{{T_{H} }}}}} \right]$$

where ZT is the dimensionless figure of merit; $T_{H}$ and $T_{C}$, are the temperature of the hotter and the cooler side, respectively. As shown in Eq. (1), the efficiency of TEG modules depends on ZT and the temperature difference maintain during TEG operation. Over the past two decades, there is significant progress in improving the ZT performance of thermoelectric (TE) materials. However, the performance of the TE modules is much lower than the theoretical efficiency due to ineffective optimization of the TEG module structure, heat losses, and electrical losses^13^.

Heat loss reduction by filling thermal insulators promises an approach to maintain a high-temperature difference of the module than improve the TEG efficiency of the module. Recently, Song et al.^1^ reported mathematical and experimental results of reducing heat loss from the TEG module using air, aerogel, Min-K, and fiberglass as thermal insulator filling material. Filling aerogel as a thermal insulator covers the modules resulting in an 8.225% efficiency improvement. Lee et al.^14^ installed a TEG module on the surface of the concrete specimen and cooling sink to maintain temperature difference and generate tiny electricity power from simulated solar light. Whalen et al.^15^ construct bismuth-telluride thermopiles and aerogel insulation which generate an average of 1.1 mW electricity power from diurnal heat flow through the soil layer of Mexico. This power range is competitive with chemical batteries. Wu et al.^16^ construct an Energy harvesting system that produces electricity from the thermal gradient across pavement structures. Multilayer insulation (MLI), multiple foils of Kapton coated with highly reflective metal and low thermal conductive spacers capable of maintaining hundreds of temperature gradients across a few millimeters thickness of insulation, was introduced for space missions in 1950^17^. Gallegos et al.^18^ Report Computational Fluid dynamics (CFD) analysis of conjugate heat transfer in multi-layer wall including an air layer for ceramic furnaces. The result shows that an air layer with a thickness of 10 cm with four partitions reduces about 44% of the heat flux through the wall concerning a single air layer with the same thickness. Multi-layer reinforced concrete slabs with thermal insulation materials are practical and widely used around the world. Several numerical research^19,20^ reported a significant reduction in energy consumption from buildings with multi-layer walls. Various computer simulation studies result in excellent agreement with the experiment^21–25^.

Since electric power capacity is widely used at approximately 200 mV/K, it becomes essential to boost the output voltage of TEGs by combining several hundred legs of the TEG module as a series circuit^26,27^. However, the conventional p/n-type module structure is complicated to produce. The p/n-type module structure creates several joining points that cause internal resistance of the TE module^26^. Typical operating environments of TEGs involve temperature fluctuations^28^ also caused thermal expansion mismatch between the p-type and n-type legs of the TEGs structure^28–30^. The unileg structure of the TEG module offers an easy-to-manufacture structure, which also reduces this thermal expansion mismatch. Moreover, this structure gives good mechanical strength and increases the lifespan of the TEG module^29^. The p/n-type TEG structure may operate at different incompatible ZT values, which harms the overall performance of the TEG module.

Oxides and perovskite TE materials have many advantages over state-of-the-art TE materials. They are cheap and abundant elements. Their high thermal and chemical stability makes it possible to use them in the air without any special coating. The versatile chemical properties and complex structures of TE oxides make it easy to modify their structure. CaMnO_3_ compound is a well-known perovskite TE material. Depending on the precursors, the synthesis process, and the microstructure, the electrical conductivity of CaMnO_3_ ranges from 10^−2^ to 6.3 S/cm at room temperature^6^.

Herein, this paper aims to investigate the effect of heat loss reduction using one-layer thermal insulator and multi-layer insulators, gaining higher temperature differences and then improving the efficiency of the TEG module. To prove the combination concept of heat loss reduction using thermal insulators and concept of direct conversion of heat loss from concrete walls generates electricity using the thermoelectric device, concrete was used as the thermal insulator covering the TEG module. A unileg n-type CaMnO_3_ TEG module without thermal insulator was measured as a control sample. The TEG module buried in concrete bricks was fabricated using a unileg structure of n-type CaMnO_3_ TEG module (called the unileg n-type CaMnO_3_ module). The CaMnO_3_ module was synthesized from the starting materials CaCO_3_ and MnO_2_ by a solid-state reaction method. The thermal insulator covering the unileg n-type CaMnO_3_ module was fabricated with varying layers of thermally insulating concrete into 2 brick-type models: 1) one layer of thermally insulating concrete (called I-layer brick) and 2) three layers of thermally insulating concrete (called III-layer brick). A computer simulation of the Finite Element Method (FEM) was used to optimize the performance of the thermoelectric concrete bricks. Thermal distribution of a CaMnO_3_ TEG modules without thermal insulator and both two types of the thermoelectric concrete bricks when applying constant temperature were investigated. Thermal and electrical behaviors when applying constant temperature were also studied both close and open circuit measurements.

## Result and discussion

### XRD, SEM and EDS characterization of CaMnO_3_ sample

XRD patterns of CaMnO_3_ sample is shown in the supplementary information (Fig. S1). The XRD patterns displayed the structural phase of the perovskite structure of the CaMnO_3_ compound, corresponding to the JCPDS# 89–0666 file. This confirmed that the CaMnO_3_ sample formed the phase of perovskite structure CaMnO_3_. Based on Rietveld refinements (goodness of fit 1.14), the calculated crystalize size of CaMnO_3_ was 2.78 μm and the calculated lattice strain was 0.022%. Additionally, the CaMnO_3_ sample was used to fabricate the unileg TEG module for the thermoelectric concrete brick.

SEM analysis and EDS mapping of the CaMnO_3_ samples are shown in the supplementary information (Fig. S2). SEM image of sintered CaMnO_3_ sample in the supplementary information (Fig. S2a) displayed a wide range of size distributions varying in size from approximately 1 to 3 μm. EDS in the supplementary information (Fig. S2b) shows the results by presenting Ca, Mn, and O atoms as an indicator of the forming phase structure of CaMnO_3_. EDS mapping of the CaMnO_3_ powder in the supplementary information (Fig. S2c) displaying a homogeneous elemental distribution of the Ca, Mn, O and C atoms on the powder surfaces. The elemental distributions of the Ca, Mn, O and C atoms on the powder surfaces of CaMnO_3_ samples were 24.74%: 38.57%: 30.73%: 5.96% for weight % ratios of Ca: Mn: O: C, and 16.52%: 18.79%: 51.41%: 13.28% for atomic % ratio of Ca: Mn: O: C, respectively. Both the weight % ratio and the atomic % ratio experimental results are closely consistent with references^30,31^.

### Thermoelectric properties and module

**Figure 1:**
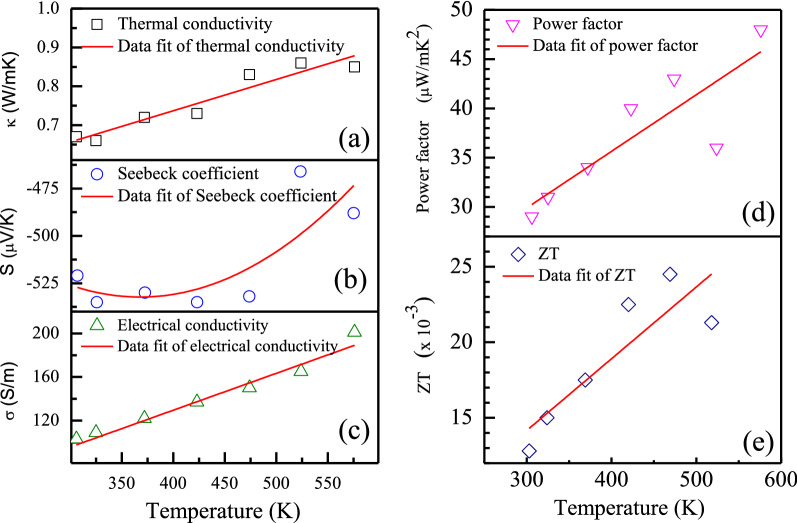
Thermoelectric properties of the CaMnO_3_ sample: (**a**) thermal conductivity, (**b**) Seebeck coefficient, (**c**) electrical conductivity, (**d**) power factor (PF), and (**e**) figure of merit (ZT) as a function of temperature.

Figure 1 shows the experimental thermal conductivity, Seebeck coefficient and electrical conductivity of the CaMnO_3_ samples as a function of the temperature between the temperature range of 300 to 600 K and the fitted curve corresponding to each data. The experimental data and the fitted curves confirm the temperature-dependence behavior of the Seebeck coefficient, electrical conductivity, and thermal conductivity. According to Fig. 1a, the thermal conductivity of the samples ranged from 0.65 to 0.85 W/mK during the temperature range of 300 to 600 K. The values of thermal conductivity slightly increased with the temperature increasing. The fitted curve corresponding to the thermal conductivity confirms the positive temperature dependence behavior of the thermal conductivity. According to Fig. 1b, the Seebeck coefficient values presented a negative sign, suggesting an n-type conductor of materials (electron is a major carrier). The Seebeck coefficient values were in the range of − 520 to  − 457 µV/K between the temperature range of 300 to 600 K. The absolute value of the Seebeck coefficient decreased with the temperature increasing. The fitted curve corresponding to the Seebeck coefficient confirms the positive temperature dependence behavior of the Seebeck coefficient. This trend indicates semiconductor behavior and the low carrier concentration of the CaMnO_3_ sample^31^. According to Fig. 1c, The electrical conductivity value was presented in the range of 100 to 200 S/m during the temperature range of 300 to 600 K. The values increased with the temperature increasing. The fitted curve corresponding to the electrical conductivity confirms the positive temperature dependence behavior of the electrical conductivity.

The calculated power factor (PF) and figure of merit (ZT) corresponding to the experimental thermal conductivity, Seebeck coefficient and electrical conductivity of the CaMnO_3_ samples during the temperature range of 300 to 600 K, as shown in Fig. 1d and e, respectively. According to Fig. 1d, the PF value was presented in the range of 29 to 50 µW/(mK^2^) during the temperature range of 300 to 600 K. According to Fig. 1e, the ZT value was presented in the range of 0.012 to 0.025 during the temperature range of 300 to 600 K. The value of both PF and ZT increased with the temperature increasing. The fitted curve corresponding to the calculated PF and ZT confirms the positive temperature dependence behavior of the PF and ZT between the temperature range of 300 to 600 K.

**Figure 2:**
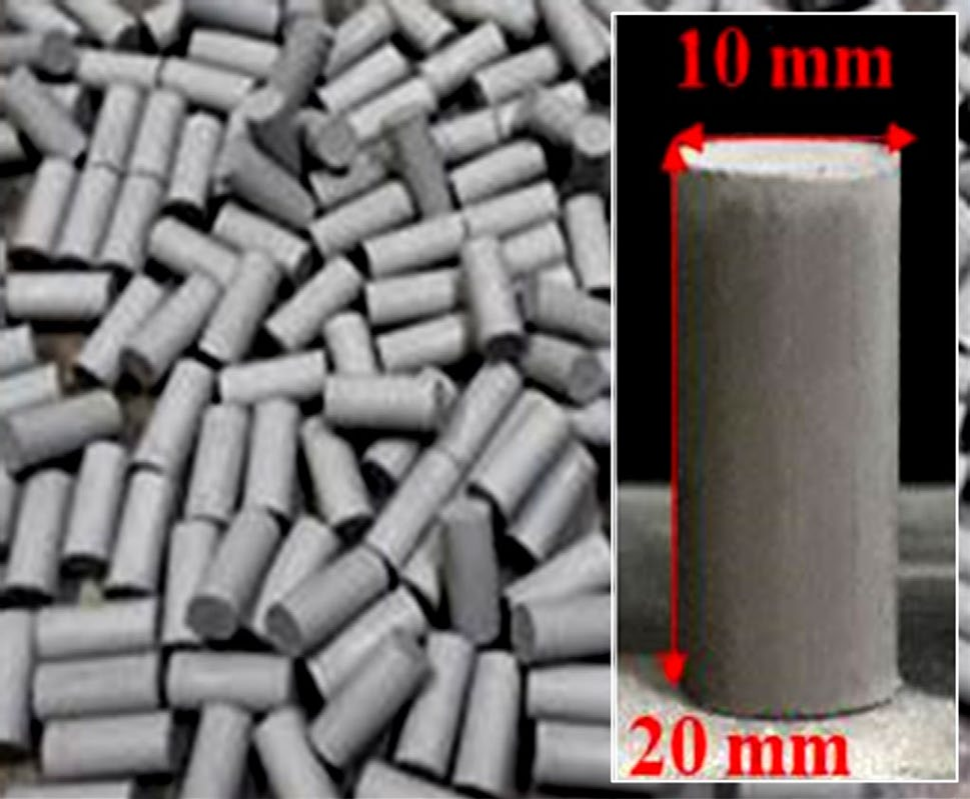
The CaMnO_3_ rods.

**Figure 3:**
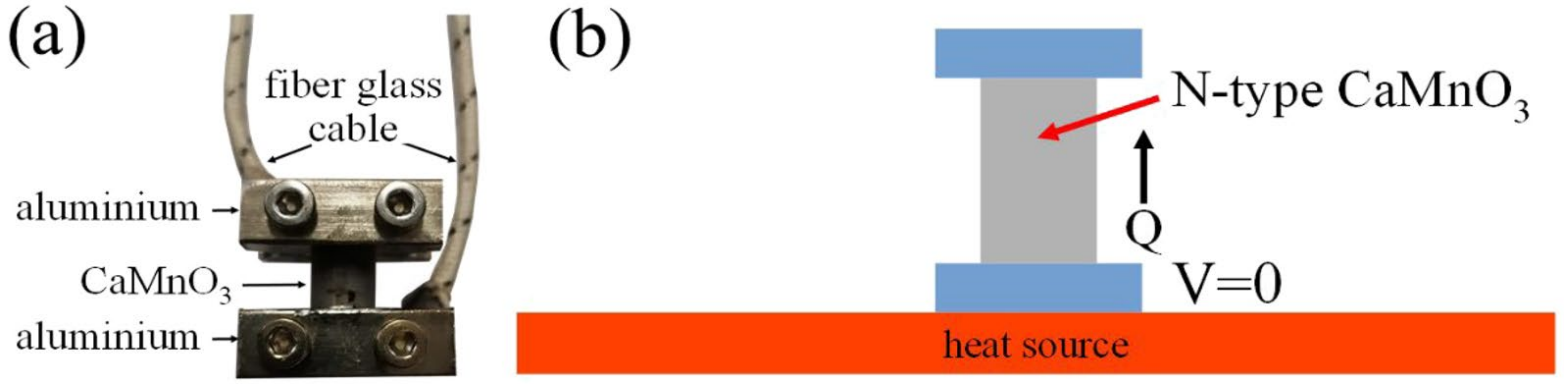
(**a**) The unileg n-type CaMnO_3_ TEG module and **(b)** schematic diagram of the unileg n-type CaMnO_3_ TEG module heated by a heat source (hot plate)

The thermoelectric rods were fabricated by using CaMaO3 powders cool pressed by a semiautonomous machine into rods with a 1.0 cm diameter, 2.0 cm vertical high and sintered by using an electric furnace at a temperature of 1100 °C for 12 h, as shown in Fig. 2. The unileg CaMnO_3_ TEG module without a thermal insulator is shown in Fig. 3a. The electrical wires, which are heat protection electrical wires, were connected with lower and upper aluminum electrodes for electrical property measurement. The lower side of the TEG module was heated by a hot plate, as shown in Fig. 3b, for thermoelectric energy conversion.

### Material properties of thermally insulating concrete

Thermally insulating concrete was used to fabricate thermoelectric concrete bricks. There are three types of cement mortar used in this work including CAST 11 LW, CAST 13 LW, and CAST 15 LW types. The chemical compositions of CAST 11 LW, CAST 13 LW, and CAST 15 LW cement mortars as provided by the commercial supplier as shown in the supplementary information (Table S1). The XRD patterns of CAST 11 LW, CAST 13 LW, and CAST 15 LW cement mortars are shown in the supplementary information (Fig. S3). The characteristic peak of the samples was indexed to Ca(OH)_2_^32–34^, Ca_2_H_0.60_ O_4.30_Si^35,36^, Ca_6_H_2_O_13_Si_3_^37,38^, SiO_2_^39^. As shown in the supplementary information (Table S2), thermal conductivities of the CAST 11 LW, CAST 13 LW and CAST 15 LW cement mortars between the temperature range of 400 to 1000 °C are temperature dependents.

### Thermoelectric concrete bricks

**Figure 4:**
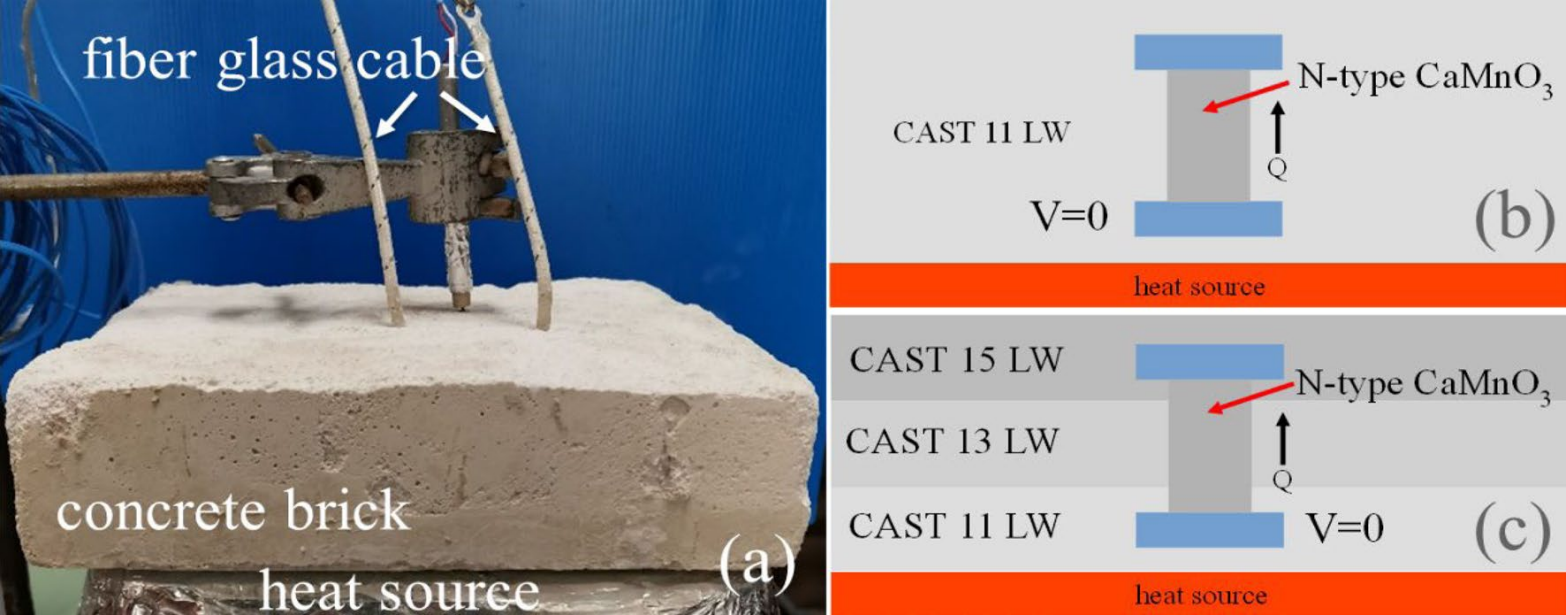
Schematic diagram of (**a**) thermoelectric concrete bricks containing the unileg CaMnO_3_ TEG module inside concrete, (**b**) the I-layer brick type (CAST 11 LW), and (**c**) the III-layer brick type (CAST 11 LW, CAST 13 LW, and CAST 15 LW).

CAST 11 LW, CAST 13 LW and CAST 15 LW are commonly used as thermal insulators of the furnace. As shown in the supplementary information (Table S2), thermal conductivity of 11 LW, CAST 13 LW and CAST 15 LW during the temperature range of 400 to 1000 between 0.25 and 0.63 W/m·K.. The thermoelectric concrete bricks shown in Fig. 4 were fabricated in two types of (1) I-layer brick and (2) III-layer brick. To archive the highest heat loss reduction, CAST 11 LW (the lowest thermal conductivity value) was fabricated in I-layer brick. Figure 4b shows the schematic diagram for the thermoelectric concrete bricks of the I-layer brick type, which use the CAST 11 LW cement mortar for thermal insulating concrete, with the unileg n-type CaMnO_3_ TEG module inside a brick. Our III-layer brick consists of three layers of different types of concrete. To increase temperature difference by increasing heat loss reduction along the vertical direction of the TEG module of III-layer brick, our three layers of concrete were designed to (1) block heat at the lowest layer, (2) release heat at the highest layer, and (3) generate thermal interface resistance at the contact of the middle layer. To obtain the highest thermal block at the lower side of the TEG module, CAST 11 LW (the lowest thermal conductivity) was fabricated at the first layer (closest layer to the heat source) of the IIII-layer brick. To generate thermal interface resistance between (1) the first layer and the second layer, and (2) the second layer and the third layer, CAST 13 LW (the middle thermal conductivity value) was fabricated as the second layer (middle layer). Finally, to archive the highest thermal release at the cooler side of the module, CAST 15 LW (the highest thermal conductivity value) was fabricated as the third layer (upper layer) of the IIII-layer brick. Figure 4c shows the schematic diagram for the thermoelectric concrete bricks of III-layer brick type which use the CAST 11 LW, CAST 13 LW and CAST 15 LW cement mortar for thermal insulating concrete, with the unileg n-type CaMnO_3_ TEG module inside a brick.

## Computational details

### Governing equations

A computer simulation of the thermoelectric concrete brick, Multiphysics® software, which is the Finite Element Method (FEM) embedded in COMSOL Multiphysics v.5.5 software^40^, was used to simulate the thermal and electrical behavior of the thermoelectric module. The governing equations using in FEM simulation as shown in the supplementary information.

### Computational model

**Figure 5:**
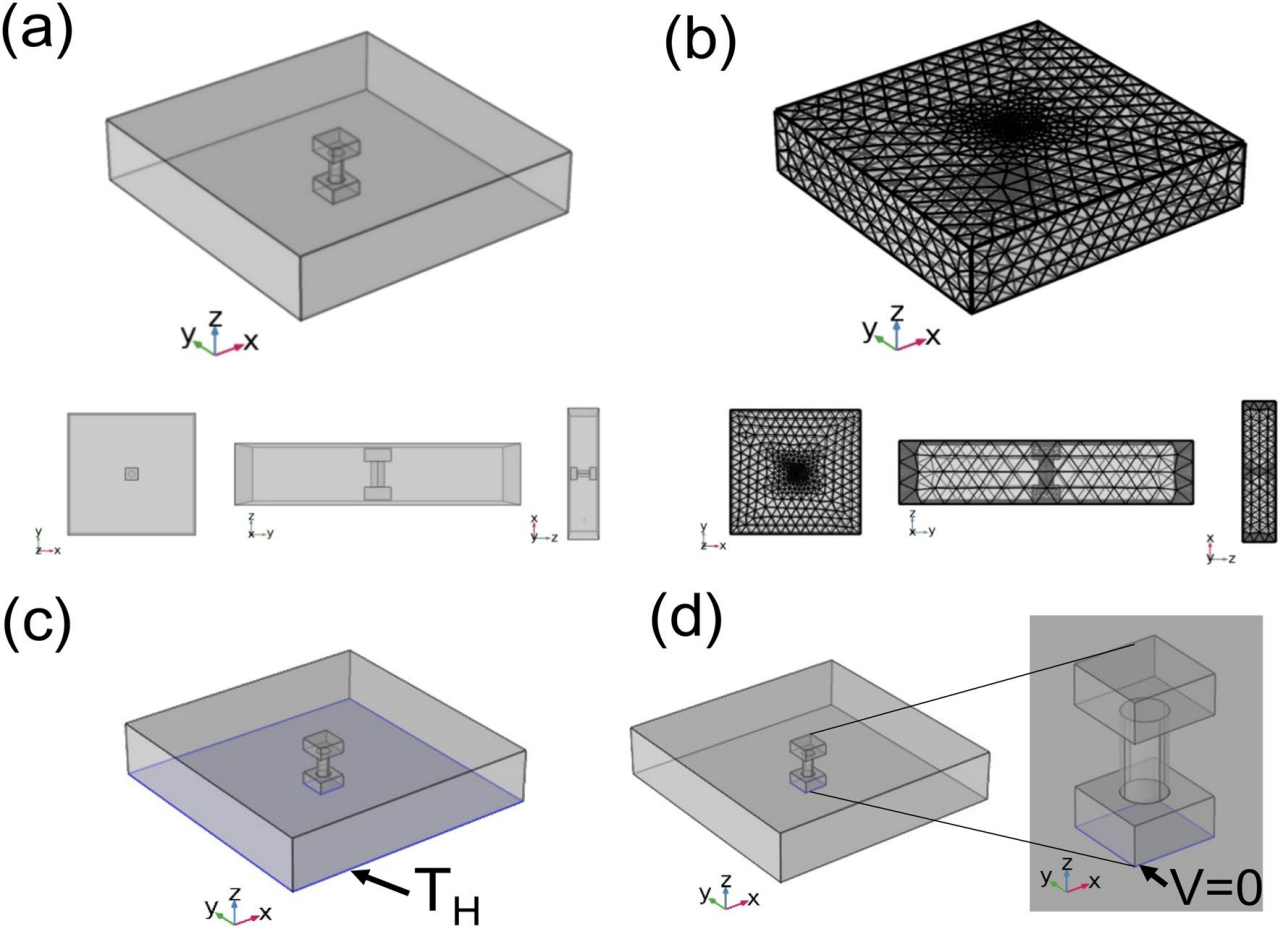
Computation model of the TEG module and the I-layer brick type of thermoelectric concrete brick using COMSOL Multiphysics v.5.5 software (https://www.comsol.com) ^40^: (**a**) Schematic diagram of the geometry model, (**b**) finite element mesh of the simulation model, and boundary condition of the model of the simulation model, (**c**) constant hotter temperature and (**d**) electric potential boundary condition at the hotter side of the TEG module and thermoelectric concrete.

The computational model of the TEG module without a thermal insulator and the I-layer brick type of the thermoelectric concrete brick are shown in Fig. 5. The CaMnO_3_ TEG module consisted of a cylindrical piece of n-type CaMnO_3_ with a diameter of 10.0 mm and a vertical length of 20.0 mm. The upper and lower sides of the CaMnO_3_ TEG module were contacted by an aluminum electrode with a size of 20.0 mm × 20.0 mm × 10.0 mm. A hot plate was used as the heat source of the CaMnO_3_ TEG module at the base position. The free electrons on the hotter temperature side (*T*_*H*_) of the CaMnO_3_ TEG module had higher kinetic energy than those on the cooler temperature side (*T*_*C*_). After that, the electron diffuses from *T*_*H*_ toward *T*_*C*_. The differential concentration of negative and positive charges along the vertical direction of the CaMnO_3_ piece due to the temperature difference between hotter and cooler temperatures (*dT* = *T*_*H*_*-T*_*C*_) caused the potential difference (*dV*). This phenomenon can be explained by Seebeck effects, Peltier effects, and Thomson effects as thermoelectric effects. The I-layer brick type of thermoelectric concrete bricks, which is the CaMnO_3_ TEG module inside a brick for one layer of CAST 11 LW concrete, was 20.0 cm × 20.0 cm × 4.5 cm in size. Figure 5a shows the schematic diagram of the geometry model used for simulation of the TEG module without the thermal insulator model and the I-layer brick type of the thermoelectric concrete brick model. Figure 5b shows the finite element mesh of the model used for simulation of the TEG module without the thermal insulator model and the I-layer brick type of the thermoelectric concrete brick model. The temperature boundary condition and potential boundary condition of the TEG module were covered by air; and, the I-layer brick type of thermoelectric concrete brick model was covered by CAST 11 LW concrete. The initial condition of the computer simulation model is room temperature and zero potential at the initial time. The boundary condition of the model consists of the constant hotter temperature at the lower side of the TEG module without the thermal insulator model and the lower side of the I-layer brick type of the thermoelectric concrete brick model, as shown in Fig. 5c**.** The electric potential boundary condition at the hotter side of the TEG module and thermoelectric concrete brick were set at zero potential (grounded), as shown in Fig. 5d. Heat current was flowing into the TEG module from the heat source only and flowing out from the TEG module at the upper electrode. Heat conduction, heat convection and heat radiation were included in the simulation of the CaMnO_3_ TEG modules. The electrical resistance from material contact was neglected.

**Figure 6:**
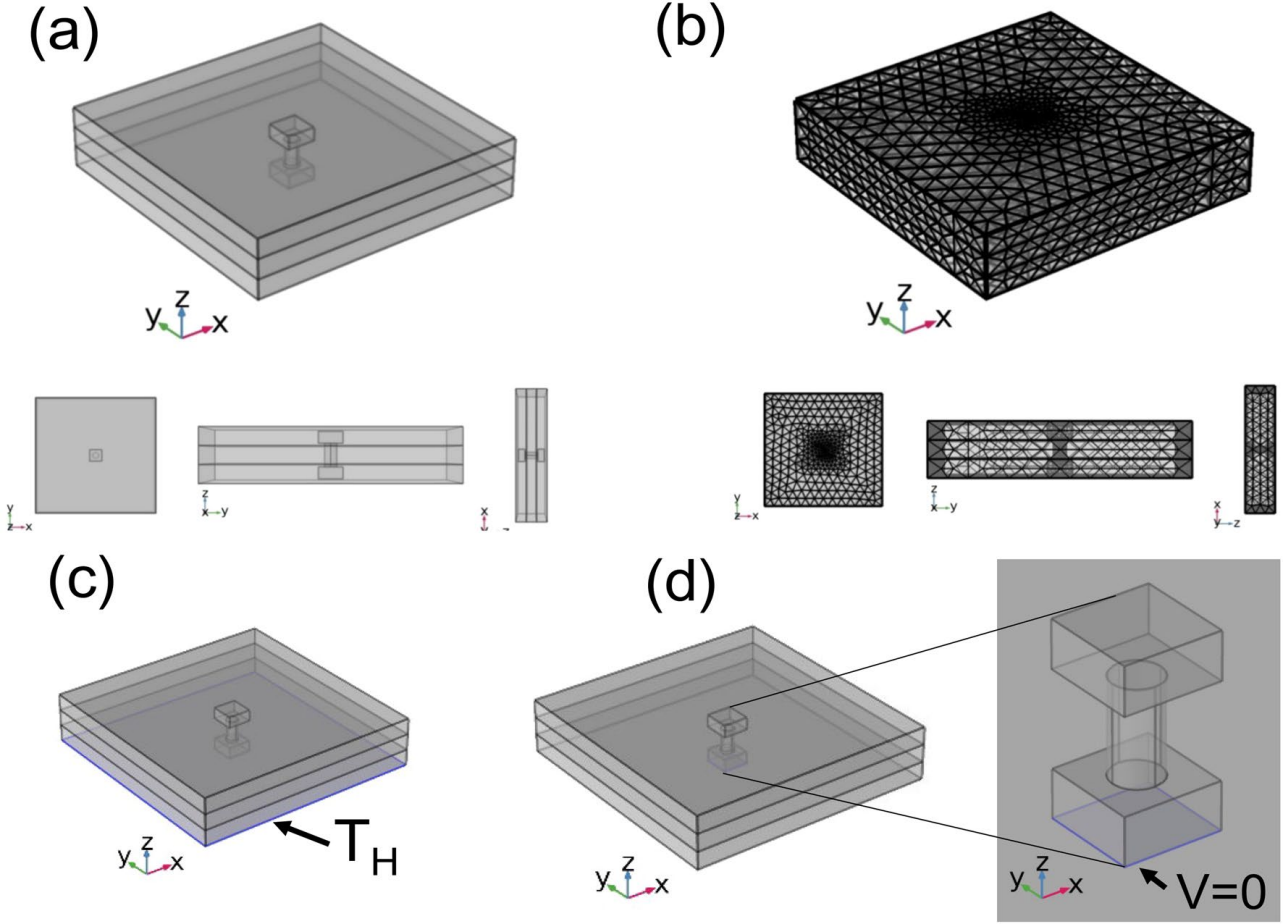
Computation model of the TEG module and the III-layer brick type of thermoelectric concrete brick using COMSOL Multiphysics v.5.5 software (https://www.comsol.com) ^40^(**a**) Schematic diagram of the geometry model, (**b**) finite element mesh of the simulation model, and boundary condition of the model of the simulation model, (**c**) constant hotter temperature and (**d**) electric potential boundary condition at the hotter side of the TEG module and thermoelectric concrete.

The computational model of the III-layer brick type of the thermoelectric concrete bricks is shown in Fig. 6a as displaying the geometry of the III-layer brick model which is the CaMnO_3_ TEG module inside a brick of three layers of CAST 11 LW, CAST 13 LW, and CAST 15 LW concrete. Figure 6b shows the finite element mesh of the III-layer brick model. The initial condition of the computer simulation model is room temperature and zero potential at the initial time. Figure 6c and d show the temperature boundary condition and potential boundary condition used in the computer simulation model of the III-layer brick type of the thermoelectric concrete bricks.

As described previously in the boundary condition, the computer simulation was assigned hotter temperature and grounded (*V* = *0*) potential at the lower side of the TEG module or the TEG module in I-layer and III-layer bricks. The computer simulation results of the temperature, temperature surface, and output voltage, as shown in the supplementary information (Fig. S4) are in good agreement with the assigned boundary conditions.

### Thermal distribution

**Figure 7:**
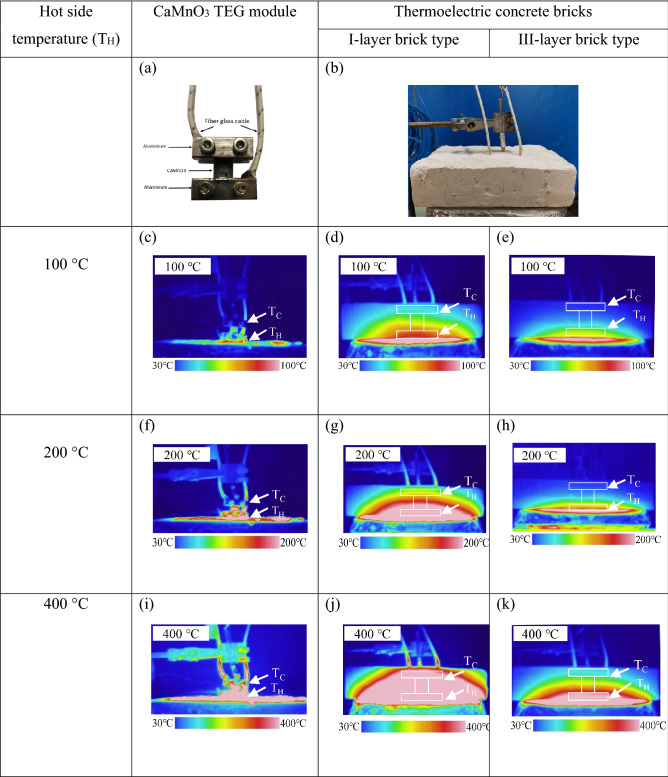
(**a**) The CaMnO_3_ TEG module without thermal insulator, (**b**) The thermoelectric concrete bricks, the IR camera image using Keysight Technologies, U5856A (https://www.keysight.com/)of (**c**) the CaMnO_3_ TEG module, and (**d**, **e**) the thermoelectric concrete bricks of the I-layer brick type and the III-layer brick type when applying the hot side temperature at 100 °C, (**d**–**f**) for temperature at 200 °C, and (**g**–**i**) for temperature at 400 °C.

Thermal Imaging Camera (Keysight Technologies, U5856A) with temperature range of − 20–650 °C was use to record infrared thermal distribution of the CaMnO_3_ TEG module without a thermal insulator and the thermoelectric concrete bricks of the I-layer brick type and the III-layer brick at hot side temperatures of 100, 200, and 400 °C are shown in Fig. 7. The results of the TEG module without a thermal insulator show a small temperature difference along the vertical direction inside the module when applying a hotter temperature of 100 °C. The results of applying hotter temperatures of 200 and 400 °C showed high temperature along the TEG module. I-layer bricks displayed the temperature difference at the hotter temperature of 100 and 200 °C, while displaying a high temperature along the TEG module during the hotter temperature of 400 °C. The results of the III-layer bricks showed temperature difference inside the TEG module along vertical direction for 100, 200 and 400 °C. Most of heat confined near the hotter side of the brick at applying the hotter temperature for 100 and 200 °C. The results indicated that the covering temperature along the I-layer and III-layer bricks were more effective than the TEG module without thermal insulator to maintain temperature difference.

**Figure 8:**
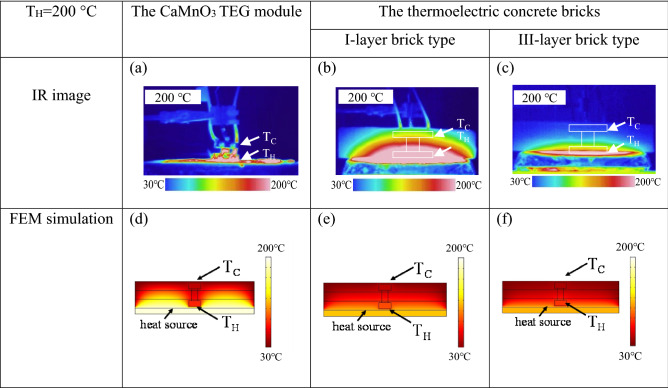
Thermal distribution image of the CaMnO_3_ TEG module without a thermal insulator and the thermoelectric concrete bricks of the I-layer brick type and the III-layer brick type upon applying a hot side temperature at 200 °C: (**a**–**c**) experimental results from the infrared image using Keysight Technologies, U5856A (https://www.keysight.com/), and (**d**–**f**) computation results from the FEM simulation using COMSOL Multiphysics v.5.5 software (https://www.comsol.com) ^40^

Figure 8 shows the thermal distribution on the CaMnO_3_ TEG module and the thermoelectric concrete bricks. The thermal distribution images of the CaMnO_3_ TEG module without the insulator, the I-layer brick, and the III-layer brick are shown in Fig. 8a, b and c, respectively. The thermal properties of the CaMnO_3_ TEG module and thermal insulating concretes upon applying a constant hotter temperature at 200 °C were recorded by an infrared camera. To validate the simulation, the FEM results were compared with the infrared images. The thermal distribution image from the time-dependent FEM simulation with applying the constant hotter temperature at 200 °C is shown in Fig. 8d, e and f**.** All experimental and FEM simulation results show a decrease in temperature along the vertical direction. Figure 8a and d show the thermal distribution images of the CaMnO_3_ TEG module without a thermal insulator. The IR image shows that the high temperature covers all parts along the vertical direction of the module. This result is also confirmed by the FEM simulation indicated by the high temperature around the module without a thermal insulator. Figure 8b and e show the experimental and computer simulation thermal distribution image of the CaMnO_3_ TEG module inside the I-layer brick. Both results show a temperature gradient along the vertical direction both inside the module and the covered thermal insulator. Figure 8c and f shows the thermal distribution image of the TEG module inside the III-layer brick from the IR image and computer simulation. Both results show a small temperature gradient along the vertical direction both inside the module and the covered thermal insulator.

### Electric conversion of thermoelectric concrete devices

**Figure 9:**
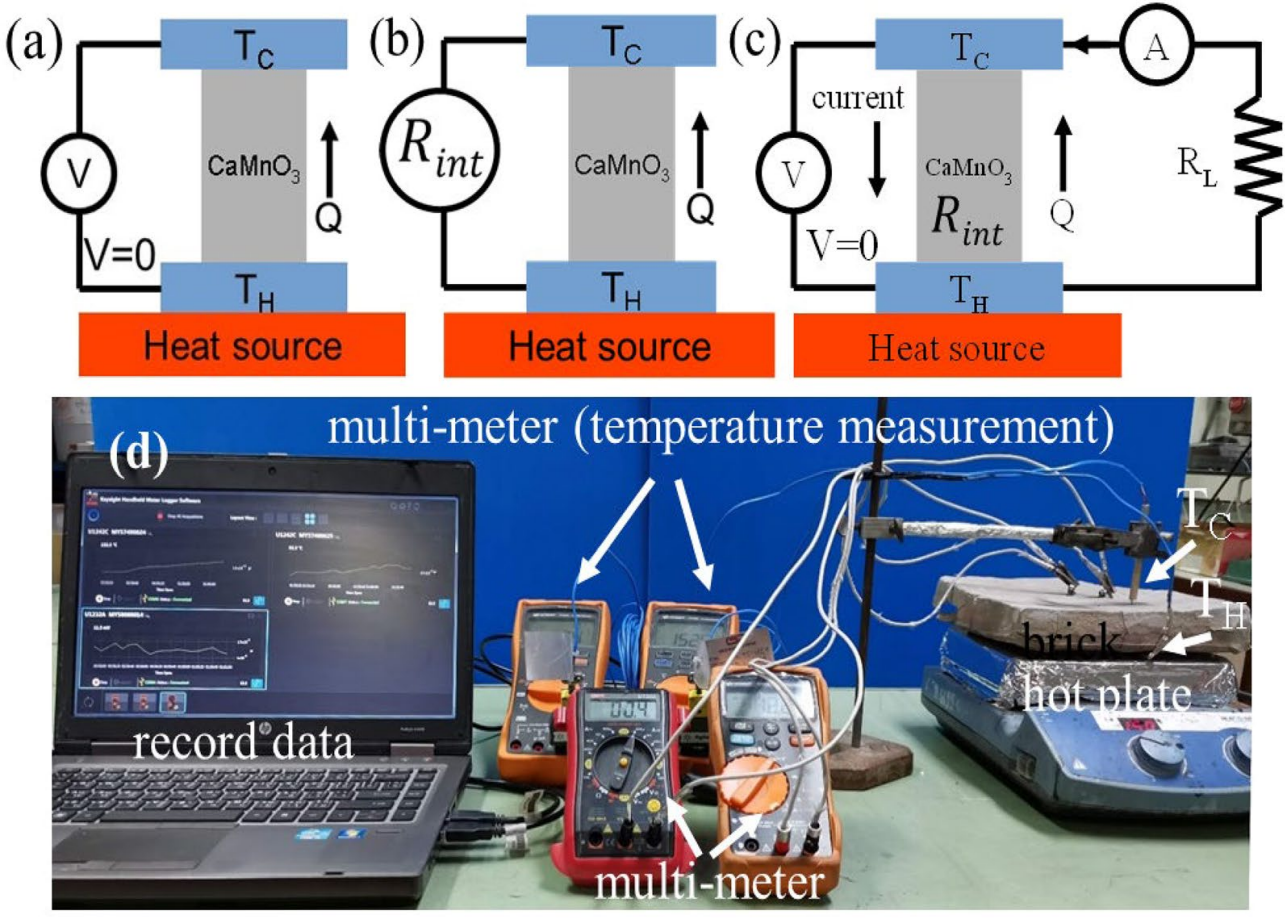
(**a**, **b**) Schematic diagram of open circuit measurement of the TEG module, (**c**) Schematic diagram of closed-circuit measurement of the TEG module, (**d**) Experimental setup of the thermoelectric concrete brick (I-layer and III-layer brick types).

The electric conversion of the CaMnO_3_ TEG module and the TEG module in I-layer and III-layer bricks were measured both open circuit and close circuit. For the TEG module without a thermal insulator, the lower side of the TEG module was heated by a hot plate. Hotter temperature (T_H_) and cooler temperature (T_C_) were measured by using multimeters with the type K thermocouple probe at the lower side and the upper side of the module, respectively. For the TEG module in I-layer and III-layer bricks, the lower side of the thermoelectric concrete brick was heated by a hot plate. Hotter temperature (T_H_) and cooler temperature (T_C_) were measured by using multimeters with the type K thermocouple probe at the lower side and the upper side of the thermoelectric concrete bricks, respectively. The temperature difference (*dT*) between the hotter temperature and the cooler temperature was calculated from *dT* = *T*_*H*_*−T*_*C*_. The TEG module without a thermal insulator and the TEG module in I-layer and III-layer bricks were connected with electrical wires and lower and upper aluminum electrodes for electrical voltage and current measurements. For open-circuit measurement, as shown in Fig. 9a, b and d, the output voltage between the lower and upper electrodes of the module was measured by using multimeter. The internal resistance was also measured by using multimeter. For closed-circuit measurement, as shown in Fig. 9c, the external electrical resistances were connected to the circuit. The output voltage was measured by using multimeter. The output current also was measured by using multimeter. All the measured data were recorded by using data logger software. For open-circuit measurement, the output voltage (dV) for the temperature difference (dT) was performed to describe the open-circuit voltage of the thermoelectric devices. Electrical properties of thermoelectric devices at constant hotter temperatures of 100, 200, and 400 °C were also measured. For close-circuit measurement, electrical properties of thermoelectric devices were characterized from I-V curve and I-P curve.

### Open-circuit measurement

**Figure 10:**
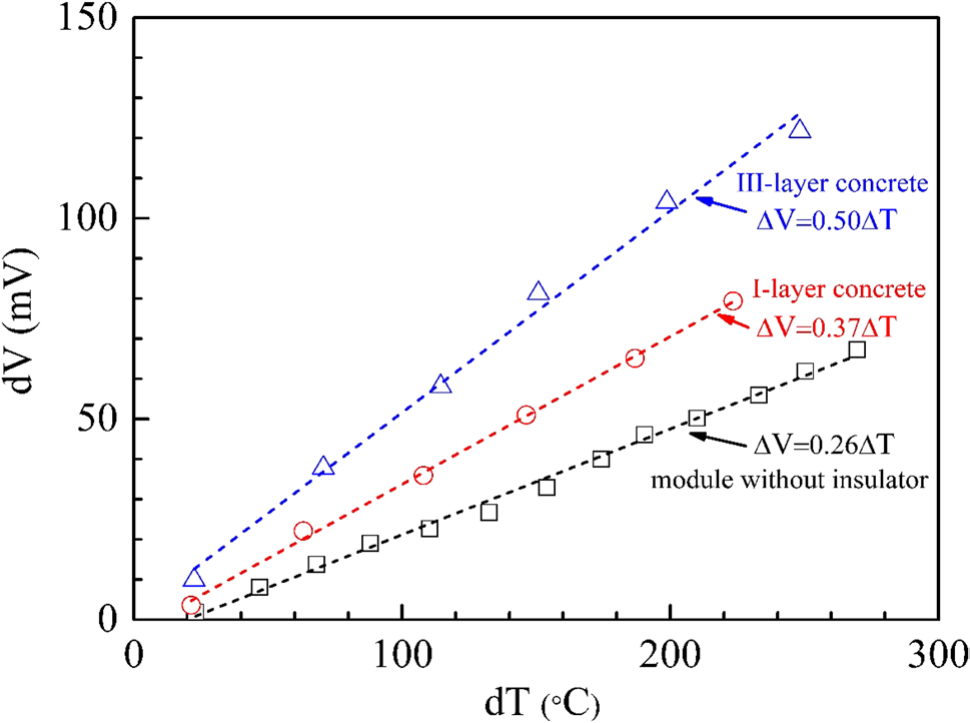
Output voltage as a function of temperature difference obtained in the open circuit measurement.

As shown in Fig. 10, the generated output voltage of the TEG module without thermal insulator, the module in I-layer brick and the module in III-layer brick were varied linearly with the temperature difference. We obtained relations $$\Delta V = 0.26\Delta T,$$$$\Delta V = 0.37\Delta T$$ and $$\Delta V = 0.50\Delta T$$ which described the open circuit voltage of the TEG module without thermal insulator, the module in I-layer brick and the module in III-layer brick, respectively.

**Figure 11:**
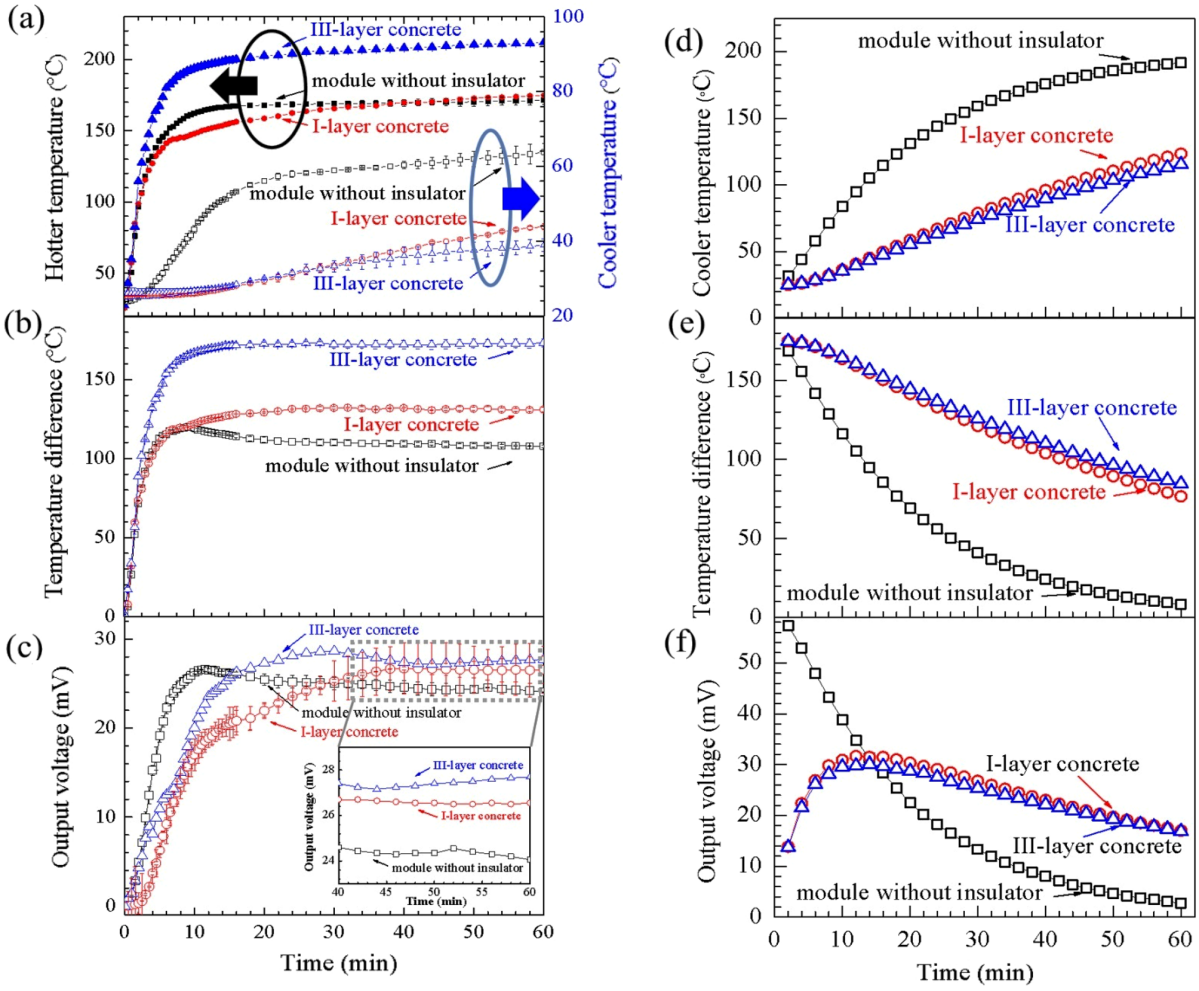
(**a**–**c**) The experimental results and (**d**–**f**) The FEM simulation results of hotter and cooler temperature, (**a**, **d**), temperature difference, (**b**, **e**) output voltage, (**c**, **f**) during the target hotter temperature of 200 °C as a function of time**.**

The experimental results of the open-circuit measurement of the CaMnO_3_ TEG module and the thermoelectric concrete brick in the I-layer brick type and III-layer brick type at a hotter temperature of 200 °C are shown in Fig. 11. The hotter temperature and cooler temperature as a function of time from the experimental results are presented in Fig. 11a. As the heat source of the experiment, a temperature control hot plate was heated starting from room temperature until reaching the target hotter temperature of 200 °C. As shown in Fig. 11a, the stable hotter temperature was achieved about 15 min later. The average hotter temperature during 40–60 min for the TEG module without a thermal insulator, the I-layer brick and the III-layer brick is 172, 172 and 210 °C, respectively. The average cooler temperature during 40–60 min for the TEG module without a thermal insulator, the I-layer brick and the III-layer brick is 62, 41 and 37 °C, respectively. The cooler temperature as a function of time from the FEM simulation results is shown in Fig. 11d**.** The results indicate that the cooler temperature from the FEM simulation results was close to that of the experimental results.

The temperature difference as a function of time from the experimental results is shown in Fig. 11b. The average temperature difference during 40–60 min for the TEG module without a thermal insulator, the I-layer brick and the III-layer brick is 108, 132 and 172 °C, respectively. As shown in the supplementary information (Fig. S5), during the hotter temperature of about 200 °C, the temperature difference of the TEG module without a thermal insulator is 108 °C and that of the I-layer brick is132 °C. This result indicated that the TEG module in I-layer brick allows a lower heat transfer (higher heat lost reduction) than the TEG module without a thermal insulator.

Considering thermal transmittance (U-value) of (1) the TEG module without thermal insulator and (2) the TEG module in I-layer brick had been calculated using the same brick geometry, as shown in the supplementary information (Fig. S6). According to the supplementary information (Fig. S6), the brick geometry consists of three parts. The lower part and the upper part are the rectangular area with the size of 20.0 cm × 20.0 cm × 1.25 cm and the middle part is the vertical rod with the size of 1.0 cm diameter and 2.0 cm vertical high inside the rectangular area with the size of 20.0 cm × 20.0 cm × 2.0 cm. The inner area of the middle part is the CaMnO3 TEG module. The total thermal resistance was calculated from the series summation of the thermal resistance of the lower, the middle and the upper parts. The thermal resistance of the middle part was calculated from the parallel summation of the thermal resistance of the inner rod and the outer area. The thermal transmittance is the reciprocal of the total thermal resistance.

Heat transfer mechanism of the TEG module without thermal insulator consist of (1) heat conduction of the CaMnO_3_ TEG rod inside the middle part and (2) heat convection of natural air at the lower, the upper and the outer area of the middle part. Firstly, the thermal resistance due to heat conduction is calculated using the equation of $R_{cond} = L/(\kappa A)$, where L is length of the TEG rod of 2.0 cm, A is cross-sectional area of 1.0 cm diameter of the TEG rod and $\kappa$ is thermal conductivity of the CaMnO_3_ TEG rod about 0.65 W/m·K. Secondly, the heat convection of natural air at the lower, the upper and the outer area of the middle part is calculated using the equation of $R_{conv} = 1/(hA)$, where h is the convection heat transfer coefficient of natural air about 25 W/m^2^·K^41,42^, A is cross-sectional area of the lower, the upper and the outer area of the middle part, respectively. The calculated total thermal resistance around 3 K/W and the thermal transmittance of the module without thermal insulator equal 8.25 W/m^2^·K.

Heat transfer mechanism of the TEG module in I-layer brick consist of (1) heat conduction of the CaMnO_3_ TEG rod inside the middle part and (2) heat conduction of CAST 11 LW as thermal insulator at the lower, the upper and the outer area of the middle part. The thermal resistance due to heat conduction of the CaMnO_3_ TEG rod is calculated using thermal conductivity of the CaMnO_3_ TEG rod equals 0.65 W/m·K. According to the supplementary information (Table S2), the values of thermal conductivity between temperature range of 400–1000 °C of CAST 11 LW cement mortar are about 0.25–0.40 W/m·K. The linear extrapolation of thermal conductivity of CAST 11 LW during the temperature range of 400 to 1000 °C had been performed and obtain the extrapolated thermal conductivity of CAST 11 LW cement mortar at 200 °C equal 0.20 W/m·K.The thermal conductivity of the CAST 11 LW equal 0.20 W/m·K is used to calculate thermal resistance of the thermally insulator covering the TEG module of the I-layer brick. The calculated total thermal resistance around 51.2 K/W and the thermal transmittance of the I-layer brick equal 0.49 W/m^2^·K.

According to the supplementary information (Fig. S6), heat transfer mechanism of the TEG module in III-layer brick consists of heat conduction of the CaMnO_3_ TEG rod inside the middle part, heat conduction of 1.25 cm length of CAST 11 LW at the lower part (R_1_), 0.25 cm of CAST 11 LW (R_2_), 1.5 cm length of CAST 13 LW (R_3_) and 0.25 cm length of CAST 15 LW (R_4_) at the outer area of the middle part, respectively and heat conduction of 1.25 cm length of CAST 15 LW at the upper part (R_5_). The thermal resistance due to heat conduction of the CaMnO_3_ TEG rod is calculated using thermal conductivity of the CaMnO_3_ TEG rod equals 0.65 W/m·K. According to the supplementary information (Table S2), the values of thermal conductivity between temperature range of 400 to 1000 °C of CAST 11 LW, CAST 13 LW and CAST 15 LW cement mortars are about 0.25 to 0.63 W/m·K. The linear extrapolation of thermal conductivity of CAST 11 LW, CAST 13 LW and CAST 15 LW during the temperature range of 400 to 1000 °C had been performed and obtain the extrapolated thermal conductivity at 200 °C are 0.20, 0.34 and 0.58 W/m·K, respectively. The extrapolated thermal conductivity of the CAST 11 LW CAST 13 LW and CAST 15 LW are applied to calculate thermal resistance of the thermally insulator covering the TEG module of the III-layer brick. The calculated total thermal resistance is about 3.69 K/W and the thermal transmittance of the I-layer brick equal 6.7 W/m^2^·K.

The calculated thermal transmittance of the TEG module without thermal insulator about 16.8 times larger than that of the I-layer brick proved concept of using thermally insulator to maintain higher temperature difference between the hotter and the cooler side of I-layer brick with respect to the TEG module without thermal insulator. The calculated thermal transmittance of the III-layer brick about 13.6 times larger than that of the I-layer brick. This calculated result contrast with the experimental temperature difference according to the supplementary information (Fig. S5). However, as reported by Grujicic et al.^43^, the effect of the thermal interface resistance must be included as a significant role in heat management of electronic devices.

The FEM simulation results are demonstrated in Fig. 11e**.** The trend of the temperature difference of all three models is in good agreement between the experiment and the simulation at a constant hotter temperature of 200 °C. The temperature difference reached the highest value near the starting time. There are temperature differences that decrease as the temperature increases to the target temperature. After that, there are constant temperature differences during the constant hotter temperature. The temperature difference of both TEG modules in I-layer and III-layer bricks had similar values and was higher than the temperature difference of the TEG module without a thermal insulator.

The output voltage as a function of time from the experimental results is shown in Fig. 11c. The average output voltage of the TEG module without a thermal insulator, the I-layer brick and the III-layer brick during 40–60 min is 24.35, 26.57 and 27.70 mV, respectively. The FEM simulation results are shown in Fig. 11f**.** The trends of both the computer simulation and experimental results are in good agreement. As a function of time and output voltage from the TEG module without a thermal insulator, the TEG module in I-layer and III-layer bricks reaches the highest output voltage between 20 and 30 mV when the temperature increases from room temperature to the target constant temperature. At a constant hotter temperature of 200 °C, there are some reductions in both the experimental and FEM simulation output voltages of the TEG module in the I-layer and III-layer bricks. At a constant hotter temperature, the output voltage of the TEG module without a thermal insulator is lower than the output voltage of the TEG module in I-layer and III-layer bricks.

As shown in Fig. 11d, e, and f, the FEM simulation results of both I-layer brick and III-layer brick display the same cooler temperature, temperature difference, and output voltage. Research by Hogblom and Andersson^25^ performed 3D finite element simulations including thermal interface resistance of commercial TEG Bi_2_TE_3_-based module. The simulation results were conducted under the same condition as in the experiments enabling excellent accurate prediction of module performance over the entries range of operation conditions. We sincerely have no proof of the FEM simulation including thermal interface resistance at this stage but we believe that the reason for our FEM results is our performed FEM simulations did not include the effect of thermal interface resistance between the lower-middle and middle-upper layer of III-layer brick.

**Figure 12:**
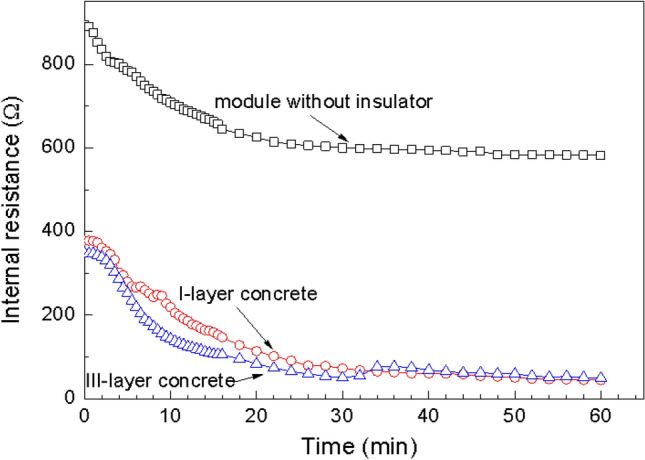
Internal resistance of the TEG module without a thermal insulator and the thermoelectric concrete brick of the I-layer and III-layer bricks as a function of time.

The internal resistance values of the TEG module without a thermal insulator and the thermoelectric concrete brick of the I-layer and III-layer bricks as a function of time are shown in Fig. 12. A schematic diagram of the open circuit internal measurement of the thermoelectric module can be seen in Fig. 4. The internal resistance of all models decreased during the beginning of the experiment while the temperature *T*_*H*_ increased from room temperature to the target constant temperature at 200 °C. The internal resistance of the module in both the I-layer and III-layer bricks had the same value of approximately 100 Ω which was lower than the internal resistance of the module without a thermal insulator of approximately 600 Ω.

According to the experimental results in Fig. 11a, the hotter side temperature was rising from starting point at room temperature until reach the target stable hotter side temperature of 200 °C after 20 min later, According to Fig. 1c, the experimental electrical conductivity of the CaMnO_3_ samples during the temperature range of 300 to 600 K is increased with the temperature increasing. According to Fig. 12, internal resistance as the reciprocal of electrical conductivity was decreased with the temperature increasing.

Based on the definition of the mean free path which is the longest distance of carrier movement without any collision. The higher mean free path indicates the higher conductivity of the sample. Internal resistance, the obstacle causes a lower mean free path of carriers. The high kinetic energy (high temperature) carriers move faster than that with lower kinetic energy and then archiving a higher mean free path. For the CaMnO_3_ TEG module, electrical carrier movement due to the different kinetic energy (difference temperature) causes the difference in the mean free path of the carrier at the hotter and the cooler side of the module. In summary, the higher difference mean free path between the carrier at the hotter side and the cooler side (due to the higher temperature difference) causes the higher electrical conductivity and the lower internal resistance of the sample.

According to Fig. 8 and Fig. 11b, during the same hotter temperature side of 200 °C, different heat transfer mechanisms caused three distinct values of the temperature difference between the hotter and the cooler side of the samples. According to the detail of the following paragraph, heat conduction is a significant part of heat transfer mechanism of (1) the TEG module without a thermal insulator, (2) the module in I-layer and (3) the module in III-layer brick. In summary, heat conduction affects temperature difference and then affects the electrical conductivity and internal resistance of the samples.

Neglect effect of thermal radiation, heat transfer mechanism of the TEG module without thermal insulator consists of heat conduction inside the CaMnO_3_ TEG module and heat convection of natural air around the TEG module. Heat transfer mechanism of the TEG module in I-layer brick are heat conduction of the CaMnO_3_ TEG module and heat conduction of the surrounding thermally insulator of CAST 11 LW with the extrapolated thermal conductivity of 0.2 W/m·K during the hotter temperature side of 200 °C. Heat transfer mechanism of the TEG module in III-layer brick consist of heat conduction of the CaMnO_3_ TEG module and heat conduction of the surrounding series of thermally insulator of CAST 11 LW (the lower part), CAST 13 LW (the middle part) and CAST 15 LW (the upper part) with the extrapolated thermal conductivity of 0.2, 0.34 and 0.58 W/m·K during the hotter temperature side of 200 °C, respectively. According to Grujicic et al.^43^, the effect of the thermal interface resistance should be included as a significant heat transfer mechanism of the TEG module in III-layer brick. These results proved concept of using thermally insulator of I-layer and III-layer brick maintain higher temperature difference between the hotter and the cooler side of the TEG module.

### Closed-circuit measurement

**Figure 13:**
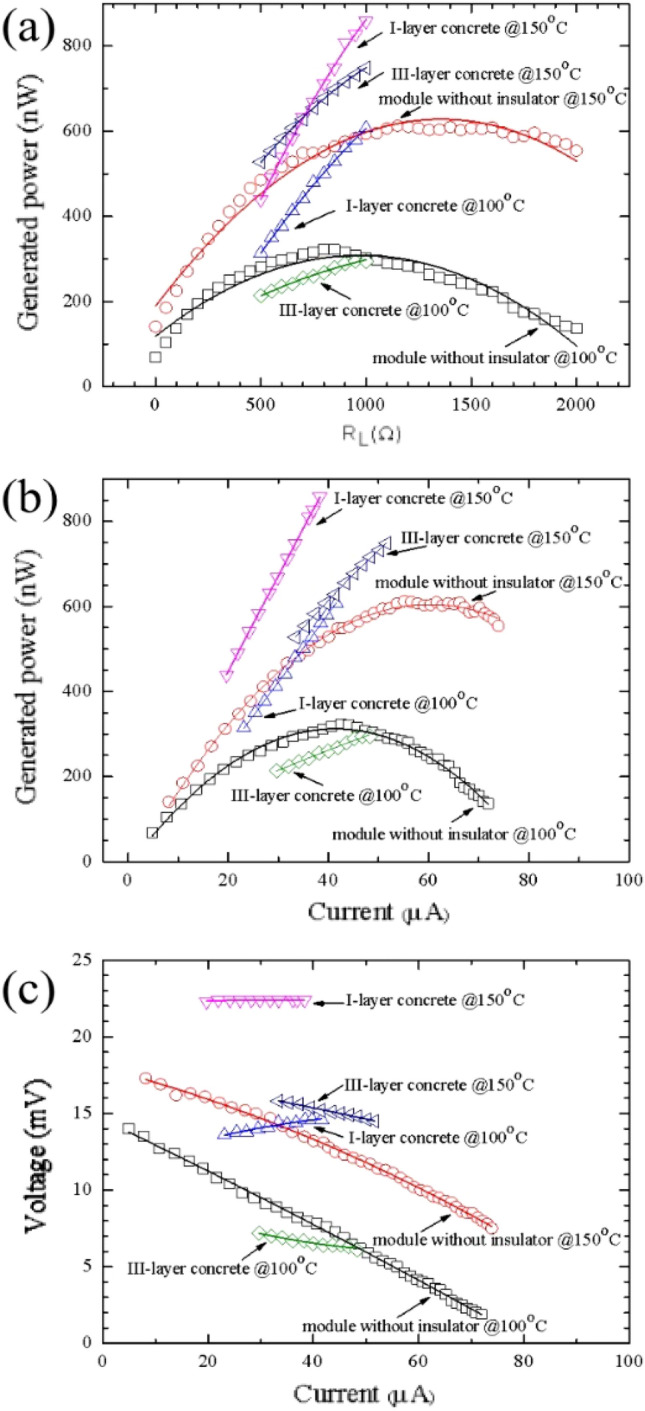
(**a**) Output power as a function of load resistance, (**b**) output power as a function of electric current, (**c**) output voltage as a function of electric current of the TEG module without an insulator, the I-layer brick and the III-layer brick.

In closed-circuit measurement, the variable external load resistance was connected to the circuit. The hotter side temperature was raised from room temperature. The hotter side, the cooler side temperature, the output voltage (V) and the output current (I) were measured and the difference temperature was calculated from *dT* = *T*_*H*_*—T*_*C*_. When the difference temperature reaches the target temperature of 100 or 150 °C, the calculated electric generation power (P = IV) as a function of load resistance between 0 and 2000 Ω of the TEG module without an insulator, between 0 and 1000 Ω of the TEG module in the I-layer and the III-layer brick are presented in Fig. 13a. The electric generation power as a function of electric current and the output voltage as a function of electric current are shown in Fig. 13b and c**,** respectively.

As shown in Fig. 13a and b, the electric generation power of the module without an insulator, the thermoelectric concrete brick of the I-layer brick and the III-layer brick at the applied difference temperatures of 100 and 150 °C displays a part of parabolic functions of the external load resistance and the electric current, respectively. The electric generation power as a function of the external load resistance and the power as a function of the electric current were increased when the difference temperature was increasing. The I-layer brick has the highest electric generation power, which is greater than the III-layer brick and the TEG module without an insulator. These results indicated that the electric generation power of the thermoelectric concrete brick for the III-layer brick type was higher than that of the I-layer brick and the TEG module. The curve of the output voltage as a function of the output current corresponding to the difference temperature of 100 and 150 °C as shown in Fig. 13c. During the difference temperature of 100 and 150 °C, our experimental results display same slope from the I-V curve of the module without thermal insulator and the module in III-layer brick. According to the literatures^44–46^, the I-V curve corresponding to each difference temperature displays the linear line with the same slope. These results indicated that the internal resistance of the module during each difference temperature had a linear behavior. However, Fig. 13c displays difference slope from the I-V curve of the module in III-layer brick. This is because there the difference rising rate of the hotter side temperature during our experiment of the module in I-layer brick.

**Figure 14:**
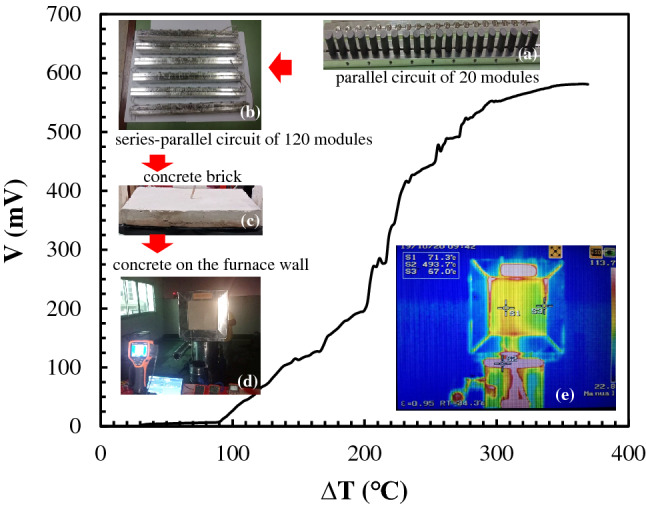
Output voltage of the 120 unileg CaMnO_3_ TEG modules in a concrete brick with embedded on a side wall of the high temperature furnace.

Direct electricity conversion from heat experiments using a TEG concrete brick embedded on a side wall of a high temperature furnace had been performed. The output voltage as a function of temperature difference between the hotter side temperature and the cooler side temperature is shown in Fig. 14. According to insertion (a) of Fig. 14, twenty modules of the unileg n-type CaMnO_3_ was connected as a parallel circuit using the upper and the lower aluminum electrodes. As shown in the insertion (b) of Fig. 14, a series–parallel combination circuit of 120 modules of the unileg n-type CaMnO_3_ was composted by using series circuit of 6 parallel circuits of the twenty modules. In the insertion (c) of Fig. 14, the TEG concrete brick was constructed by using I-layer concrete as thermal insulator for covering the series–parallel combination circuit of 120 modules of the unileg n-type CaMnO_3_. According to the heat source inside the furnace, the outer side of the furnace wall were performed as the hotter temperature side for supplying heat to the TEG concrete brick. By the insertion (d) of Fig. 14, the TEG concrete brick was embedded on the outer surface of the furnace for linking to the heat source from the furnace. According to Fig. 14, at maximum temperature of 580 °C from the hotter side temperature of the concrete brick, the temperature difference between the hotter side and the cooler side of the brick occurred at 365 °C. The maximum output voltage was obtained 581.7 mV. In the insertion (e) of Fig. 14, the thermal distribution images by infrared thermal imaging camera of the concrete brick by embedded on surface wall of the furnace as indexing by S1 (71.3 °C) and S3 (67.0 °C) displayed the cooler side temperature of the concrete brick. The thermal distribution images by infrared thermal imaging camera of the open end as indexing by S2 (493.7 °C) also exhibited the hotter side temperature of the TEG concrete brick as close to the heat source temperature from the furnace.

## Conclusion

Thermoelectric concrete bricks that effectively block heat and convert waste heat directly into electric power were designed and constructed by the unileg n-type CaMnO_3_ TEG module, the thermoelectric concrete bricks of the I-layer and III-layer bricks. The temperature difference result indicates the higher efficiency of maintaining the temperature difference along the vertical direction of the TEG module in the I-layer and III-layer bricks instead of the CaMnO_3_ TEG module without a thermal insulator. The output voltage predicts a higher performance to convert waste heat directly into electricity of both thermoelectric concrete bricks of the I-layer and III-layer bricks than the CaMnO_3_ TEG module without a thermal insulator. The trends of the temperature difference and the output voltage of the experimental and computer simulations are similar. The temperature value and output potential value of the experiment and the computer simulation are still slightly different. The III-layer brick type displayed high-performance electric generation power. Additionally, these materials displayed high performance of thermoelectric concrete brick in the III-layer brick model in electric generation due to the temperature difference.

## Method

### Preparations

CaMnO_3_ powder was synthesized by a solid-state reaction method using starting materials from commercial CaCO_3_ (99% purity Sigma–Aldrich) and MnO_2_ (99% purity Sigma–Aldrich). The starting powders were weighed in stoichiometric amounts, mixed together by using the ball milling method and cool pressed by a homemade semiautonomous machine, as shown in the supplementary information (Fig. S8), into rods with a 10.0 mm diameter and 20.0 vertical. The thermoelectric rods were sintered by using an electric furnace at a temperature of 1373 K for 12 h.

### Characterizations

The synthesis phases of the CaMnO_3_ samples were characterized by powder XRD using a PHILIPS X’ Pert MPD diffractometer with Cu Ka radiation in the range of 10–80 °C. Scanning electron microscopy (*JEOL* SEM *JSM-5800 LV)* was used to observe the morphologies and grain sizes of the CaMnO_3_ samples and to determine the homogeneous distribution of atoms on the CaMnO_3_ powder surfaces by energy dispersive X-ray spectroscopy (EDX mapping). The Sebeck coefficient and electrical resistivity were simultaneously carried out on a sample bar using an.

LSR -3 Linseis Seebeck Coefficient & Electric Resistivity Unit from Linseis Inc. The specific heat and thermal conductivity were measured on flat samples of approximately 10-mm diameter and 2–3 mm thickness using a NETZSCHLFA 477 Nano-Flash thermal diffusivity analyzer.

### Construction of thermoelectric modules

A unileg CaMnO_3_ TEG module, as shown in Fig. 3, was constructed from a cylindrical piece of a CaMnO_3_ rod with a 10.0 mm diameter and 20.0 vertical length. Both the lower and upper parts of the thermoelectric piece were covered by an aluminum electrode size of 10.0 mm × 20.0 mm × 20.0 mm. The unileg CaMnO_3_ TEG module without a thermal insulator is shown in Fig. 3a. The electrical wires, which are heat protection electrical wires, were connected with lower and upper aluminum electrodes for electrical property measurement. The lower side of the TEG module was heated by a hot plate, as shown in Fig. 3b.

### Fabrication of the thermoelectric concrete brick

The unileg n-type CaMnO_3_ TEG module (CaMnO_3_ TEG module) was applied for the construction of thermoelectric concrete bricks. Thermoelectric concrete bricks were fabricated by burying the CaMnO_3_ TEG module inside concrete, as shown in Fig. 4a**.** The concrete casting brick was prepared with dimensions of 20.0 cm × 20.0 cm × 4.5 cm. According to Fig. 4b and c, the concrete brick was produced into the I-layer brick type and the III-layer brick type. Then, the CaMnO_3_ TEG module was contained at the middle of the concrete casting brick wrapped in plastic. Next, the concrete bricks were heated to dry the concrete and melt the plastic wrap by step-up heating from room temperature to 350 °C in 24 h.

Through the above process, thermoelectric concrete bricks were fabricated in two types: 1) One layer of concrete brick (I-layer brick) and 2) three layers of concrete brick (III-layer brick). The I-layer brick type was the thermoelectric concrete brick type obtained by fabrication using the CaMnO_3_ TEG module covered by one layer of CAST 11 LW concrete. The chemical composition of CAST 11 LW cement mortar type is shown in the supplementary information (Table S1). It was used for thermal insulating castable brick (ASTM C 401 Class 0) with a extrapolated thermal conductivity of 0.20 W/mK at the temperature of 200 °C. Figure 4b shows a schematic diagram of thermoelectric concrete bricks of the I-layer brick type.

In addition, the thermoelectric concrete brick of the III-layer brick type was fabricated using the CaMnO_3_ TEG module covered by three layers of CAST 11 LW, CAST 13 LW, and CAST 15 LW concrete. The chemical composition of CAST 13 LW and CAST 15 LW cement mortar types are shown in the supplementary information (Table S1). They were used for thermally insulating castable brick in ASTM C 401 Class Q for CAST 13 LW and ASTM C 401 Class S for CAST 15 LW. The CAST 13 LW and CAST 15 LW cement had extrapolated thermal conductivities of 0.34 W/mK and 0.58 W/mK at the temperature of 200 °C, respectively. Figure 4c shows a schematic diagram of thermoelectric concrete bricks of the III-layer brick type. The arrangement of cement mortar for the thermoelectric concrete bricks was arranged by high to low values of thermal conductivity from 0.20 W/mK of CAST 11 LW, to 0.34 W/mK of CAST 13 LW) and to 0.58 W/mK of CAST 15 LW from bottom to top of the bricks.

### Characterization of thermoelectric devices

The electric conversion of the CaMnO_3_ TEG module and the TEG module in I-layer and III-layer bricks were measured both open circuit and close circuit. For the TEG module without a thermal insulator, the lower side of the TEG module was heated by a hot plate. Hotter temperature (T_H_) and cooler temperature (T_C_) were measured by using 4digit multimeters (KEYSIGHT Technologies, U1242C) with the type K thermocouple probe at the lower side and the upper side of the module, respectively. For the TEG module in I-layer and III-layer bricks, the lower side of the thermoelectric concrete brick was heated by a hot plate. Hotter temperature (T_H_) and cooler temperature (T_C_) were measured by using 4digit multimeters (KEYSIGHT Technologies, U1242C) with the type K thermocouple probe at the lower side and the upper side of the thermoelectric concrete bricks, respectively. The temperature difference (*dT*) between the hotter temperature and the cooler temperature was calculated from *dT* = *T*_*H*_*—T*_*C*_. The TEG module without a thermal insulator and the TEG module in I-layer and III-layer bricks were connected with electrical wires and lower and upper aluminum electrodes for electrical voltage and current measurements. For open-circuit measurement, as shown in Fig. 9a and b, the output voltage between the lower and upper electrodes of the module was measured by using 3.5 digits multimeter (KEYSIGHT Technologies, U1232A). The internal resistance was also measured by using 3.5 digits multimeter (KEYSIGHT Technologies, U1232A). For closed-circuit measurement, as shown in Fig. 9c, the external electrical resistances were connected to the circuit. The output voltage was measured by using 3.5 digits multimeter (KEYSIGHT Technologies, U1232A). The output current was measured by using digital multimeter (UNI-T, UT30A). All the measured data were recorded by using Keysight Handheld Meter Logger Software. The infrared thermal imaging camera (Keysight Technologies U5856A) was used for observing the surface temperature distribution.

For open-circuit measurement, the output voltage (dV) for the temperature difference (dT) was performed to describe the open-circuit voltage of the thermoelectric devices. Electrical properties of thermoelectric devices at constant hotter temperatures of 100, 200, and 400 °C were also measured. For close-circuit measurement, electrical properties of thermoelectric device were characterized from I-V curve and I-P curve.

## Supplementary Information

Supplementary Information.
