# Supplementary material for: Fabrication and thermoelectric conversion of thermoelectric concrete brick with buried unileg N-type CaMnO3 thermoelectric module inside
Source: Sci Rep. 2023 Jan 17;13:916. doi: 10.1038/s41598-023-28080-7 (PMC9845234; doi:10.1038/s41598-023-28080-7)
Supplement: Supplementary file 1 — Supplementary Information. [file 41598_2023_28080_MOESM1_ESM.docx]

**Fabrication and Thermoelectric Conversion of Thermoelectric Concrete Brick with Buried Unileg N-Type CaMnO_3_ Thermoelectric Module Inside**

**Keerati Maneesai^1,2,3,4^, Sunisar Khammahong ^1,2,3,4^, Pongsakorn Siripoom^1,2^****,**

**Chaiwat Phrompet^2,3,4,7^, Chaval Sriwong^2,3,4,6^, Santi Maensiri^5^ and Chesta Ruttanapun^1,2,3,4^,***

^1^Department of Physics, School of Science, King Mongkut’s Institute of Technology Ladkrabang, Chalongkrung Road, Ladkrabang, Bangkok 10520, Thailand

^2^Smart Materials Research and Innovation Unit, School of Science, King Mongkut’s Institute of Technology Ladkrabang, Chalongkrung Road, Ladkrabang, Bangkok 10520, Thailand

^3^Center of Excellence in Smart Materials Research and Innovation, King Mongkut’s Institute of Technology Ladkrabang, Chalongkrung Road, Ladkrabang, Bangkok 10520, Thailand

^4^Thailand Center of Excellence in Physics, Ministry of Higher Education, Science, Research and Innovation, 328 Si Ayutthaya Road, Bangkok 10400, Thailand

^5^School of Physics, Institute of Science, Suranaree University of Technology, Nakhon Ratchasima 30000, Thailand

^6^Department of Chemistry, School of Science, King Mongkut’s Institute of Technology Ladkrabang, Chalongkrung Road, Ladkrabang, Bangkok, 10520, Thailand

^7^College of Innovation and Industrial Management, King Mongkut’s Institute of Technology Ladkrabang, Chalongkrung Road, Ladkrabang, Bangkok, 10520, Thailand

**Supplemental information**





Fig. S1 XRD patterns of the CaMnO_3_ samples corresponding to data from the
JCPDS# 89-0666 file for perovskite structure.

(a)


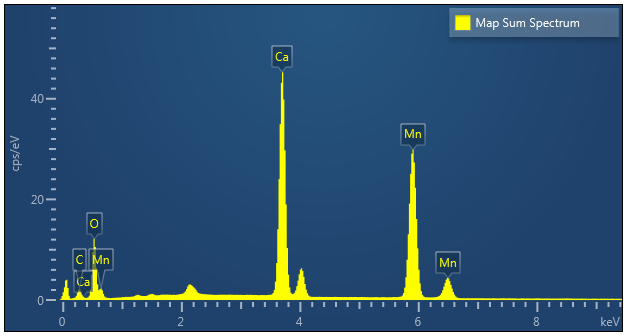
n

(b)


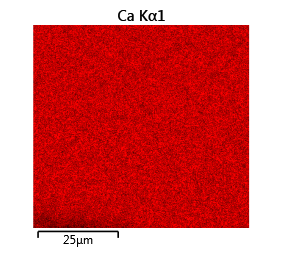

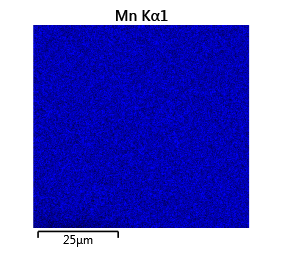

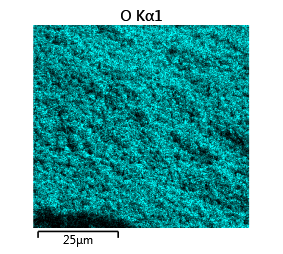

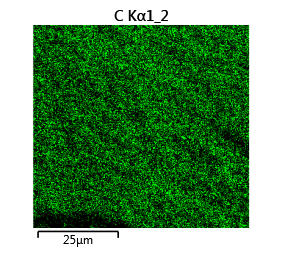


(c)

Fig. S2 (a) SEM image of sintered CaMnO_3_ powder, (b) EDS results and (c) elemental distribution by EDS mapping of the CaMnO_3_ sample.

**Fig. S3** XRD patterns of the CAST 11 LW, CAST 13 LW, and CAST 15 LW cement mortars for thermally insulating concrete.”

**Table S1** Chemical compositions of the CAST 11 LW, CAST 13 LW and CAST 15 LW cement mortars.

| Chemical composition (%) | CAST 11 LW | CAST 13 LW | CAST 15 LW |
| --- | --- | --- | --- |
| Alumina (Al_2_O_3_) | 14.5 | 41.0 | 66.0 |
| Silica (SiO2) | 54.5 | 42.0 | 15.0 |
| Iron oxide (Fe2O3) | 0.5 | 1.5 | 1.5 |
| Lime (CaO) | 29.0 | 12.5 | 15.0 |

**Table S2** The material properties of the thermally insulating concretes of the CAST 11 LW, CAST 13 LW and CAST 15 LW cement mortars.

| Thermal properties | CAST 11 LW | CAST 13 LW | CAST 15 LW |
| --- | --- | --- | --- |
| Specific heat (J/kg·K) | 880 | 880 | 880 |
| Density (kg/m^3^) | 1025 | 1400 | 1500 |
| Thermal conductivity at 400°C (W/m·K) | 0.25 | 0.36 | 0.60 |
| Thermal conductivity at 600°C (W/m·K) | 0.30 | 0.38 | 0.60 |
| Thermal conductivity at 800°C (W/m·K) | 0.34 | 0.39 | 0.61 |
| Thermal conductivity at 1000°C (W/m·K) | 0.40 | 0.42 | 0.63 |

**Governing equations**

The governing equations of the time-dependent (transient) heat transfer mechanisms were used to describe the model in this work as follows:

$\rho C_{p}u\cdot\nabla T+\nabla\cdot q=Q+Q_{ted},q=-k\nabla T$ (1),

where $\rho$ is the density (g/cm^3^); $C_{p}$is the specific heat at constant pressure (kJ/kg·K); *T* is the absolute temperature (K); *q* is the heat flux in conduction; *Q* is the heat source (W); *Q_ted_* is the thermoelastic effect (W); and, *k* is the thermal conductivity (W/m·K). The conservation of the electric current equation was used to calculate the electric current, electric field, and potential difference in the model as follows:

$\nabla J=Q_{j},\mathrm{J}=\sigma E+J_{e},\mathrm{E}=-\nabla V$ (2),

where *J* is the induced electric current (A/m^2^); *Q_j_* is the current source (W); $\sigma$is the electrical conductivity (S/m); *E* is the electric field (V/m); *V* is the electric potential (V); and, *J_e_* is the external current source (A/m^2^). A couple of equations between electric current and heat transfer in the material were described by the Seebeck, Peltier, and Thomson effect relation as follows:

$q=PJ,P=ST,J_{e}=-\sigma S\nabla T$ (3),

where *P* is the Peltier coefficient (W/A) and *S* is the Seebeck coefficient (μV/K) [1].

**Validation of models**

As described in the boundary condition, the computer simulation was assigned hotter temperature and grounded (*V=0*) potential at the lower side of the TEG module or the TEG module in I-layer and III-layer bricks. The computer simulation results of the temperature, temperature surface, and output voltage, as shown in Fig. S4(a)-(i), are in good agreement with the assigned boundary conditions. Fig. S4 (a), (d) and (g) show the temperature contours and Fig. S4(b), (e) and (h) show the temperature contours of the TEG module and the TEG module in I-layer and III-layer bricks, respectively. The hotter temperature was obtained at the lower side and the lower cooler temperature was obtained at the upper side. As shown in Fig. S4(c), (f) and (i), the output voltage result shows a negative potential at the upper side of the module due to high-energy electrons diffusing from the lower side to the upper side of the module.

Fig. S4 Computer simulation results using COMSOL Multiphysics v.5.5 software (https://www.comsol.com) [2] for (a) the temperature, (b) the temperature contour, (c) the output voltage of the unileg CaMnO_3_ TEG module, (d),(e),(f) of the TEG module in the I-layer brick type and (g),(h),(i) of the TEG module in the III-layer brick type.

Fig.S5 Schematic diagram of heat loss reduction by thermal insulator and thermal interface resistance to maintain higher temperature difference.

Fig. S6 The brick geometry used during thermal transmittance calculation of (a) the TEG module without thermal insulator and the module in I-layer brick and (b) the module in III-layer brick.

Constant external resistances of 15, 150 and 1500 Ω were applied to load resistances in the closed-circuit measurement of the unileg n-type CaMnO_3_ TEG module without a thermal insulator at a temperature difference of 100 and150 °C. The electric current and output voltage of the closed circuit were measured for 60 minutes. As shown in Fig. 6, the electric generation power (*P*) of the TEG module as a function of time was calculated by multiplying the electrical current (*I*) and the voltage (*V*) by *P=IV*. All the results showed a peak of the electric power between 5 and 20 minutes when climbing the hotter temperature from room temperature to the targeted temperature difference of 100 or 150 °C. After that, there was a small reduction and constant generated power when applying the constant temperature difference of 100 or 150 °C.

To compare the results of the external load resistance of 15, 150 and 1500 Ω, the highest constant electric power was approximately 1800 nW when using a 1500 Ω external load. These results confirmed the internal resistance of the TEG module without thermal resistance at approximately 1500 Ω.

Fig. S7 Electric generation power as a function of time of the TEG module without a thermal insulator with constant external resistances of 15, 150 and 1500 Ω.


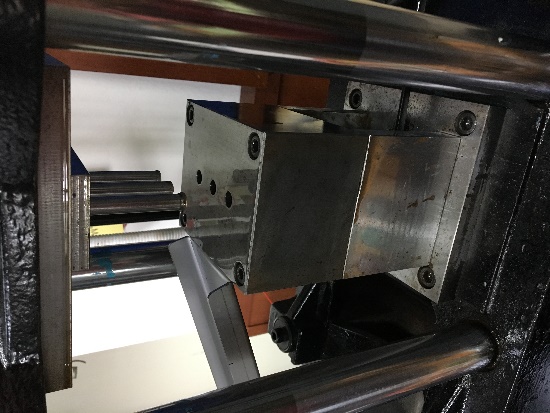

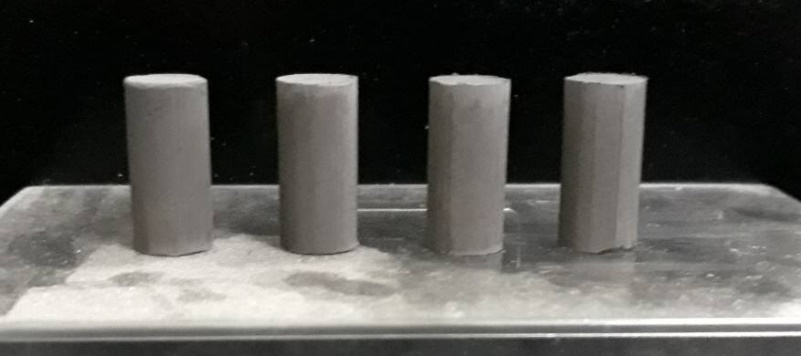


(a) (b)

Fig. S8 (a) Semiautonomous machine cool pressing into rod shape, (b) thermoelectric rods.

**References**

1. Al Musleh, M., et al., *Thermoelectric generator characterization at extra-low-temperature difference for building applications in extreme hot climates: Experimental and numerical study.* Energy and Buildings, 2020. **225**: p. 110285.

2. *COMSOL Multiphysics® v.5.5*. COMSOL AB, Stockholm, Sweden.
